# Dedication to Mingjia Dai, Ph.D. for Discovery of the First Successful Treatment of the Mal de Debarquement Syndrome

**DOI:** 10.3389/fneur.2019.01196

**Published:** 2019-12-13

**Authors:** Bernard Cohen

**Affiliations:** Department of Neurology, Icahn School of Medicine at Mount Sinai, New York, NY, United States

**Keywords:** vestibular, vestibulo-cerebellar, motion sickness, velocity storage, nodulus

## Introduction

It is with deep sadness that we note the passing of Mingjia Dai, Ph.D. Assistant Professor of Neurology on February 7th, 2019, from esophageal and lung cancer. He was 71 years old.

Dai grew up in Guangzhou, China, and completed his Ph.D. in Sydney, Australia with Drs. Michael Halmagyi and Ian Curthoys. In 1989, he came to Mount Sinai to work with Dr. Cohen. Dai was an innovative, imaginative scientist who loved working with patients. He dedicated the later part of his life to studying a rare neurological disorder, the Mal de Debarquement Syndrome (MdDS). His warmth and ability to establish relationships with patients affected by MdDS made him famous in the US and all over the world.

## Background

In 1979, Drs. Raphan and Cohen at Mount Sinai (1) and Robinson at John's Hopkins (2) discovered that a process in the vestibular nuclei functioned to extend the angular velocity-related information generated in the semicircular canals. Raphan and Cohen labeled the process as a Velocity Storage Integrator, since it described the dynamic integration of the head rotation velocity. They characterized it by the time constants of the cellular activity at the beginning and end of rotation. A few years later, with Dai, they discovered that Velocity Storage acted as a gyroscope to orient the axis of eye rotation to the spatial vertical, regardless of the position of the axis of body rotation (3, 4). This led to the discovery that Velocity Storage was an integral part of the process that produced motion sickness (5), and that susceptibility to motion sickness fell along with reductions in the time constant of Velocity Storage (6). Following this with data from Solomon and Wearne that Velocity Storage was controlled by the nodulus of the vestibulo-cerebellum (7–10), they found that motion sickness could be extinguished by repeated vestibular testing that shortened the vestibular time constant (5, 6, 11).

## Dai's Studies That Determined the Cause of the Mal de Debarquement Syndrome

In 2009, based on experiments in monkeys using an unusual paradigm, continuous rotation in darkness, coupled with side to side roll (roll while rotation, RWR), Dai had a brilliant insight. He reasoned that the prolonged exposure to this maneuver had produced some of the essential clinical signs in the monkey observed in patients with MdDS (12). Namely, there was positional nystagmus with upward quick phases when the head was rolled to one side. From it, he postulated that roll while rotation had caused a shift in the “eigen vector” in roll, i.e., the central representation of gravity, and that MdDS might be the result of this shift in the roll vector (13). In other experiments, neither spontaneous vestibular nystagmus, nor unusual positional nystagmus occurred in monkeys with a very short vestibular time constant, confirming that the Velocity Storage Integrator had played a critical role in producing MdDS (1, 13).

## Dai's Treatment of the MdDS

Dai then devised a treatment to reverse this vestibular maladaptation. He rolled the head back and forth at the frequency of the perceived body oscillations, while watching a slowly moving full field optokinetic stimulus moving against the vestibular imbalance as determined by the Fukuda Stepping Test (14). The OKN reoriented Velocity Storage back to the spatial vertical (15). Remarkably, the sensed body oscillations were 0.2 Hz after the sea voyages in almost all of the MdDS sufferers, indicating that the MdDS was being generated by a disordered rhythm, likely in the vestibular and vestibulo-cerebellar systems (13).

A critical aspect of the treatment was that the direction of the optokinetic stimulus had to be opposite to the direction of a vestibular imbalance that had been produced during the roll while rotation or by travel on the sea. If the movement of the OKN stripes was in the direction of the vestibular imbalance determined by the Fukuda Stepping Test, the MdDS patients became worse, and, as shown by Mucci et al. (16) if the OKN stripes did not move, the patients had no improvement.

Of note, the primary findings were in the body and limbs and not in the eyes in the Vestibulo-Ocular Reflex (VOR), and there was only rarely spontaneous nystagmus in these patients. This series of maneuvers is described in detail in a publication (of which Dai was the first author) in 2014 (17).

## Results

With the Dai et al. treatment, there was an immediate improvement rate of 75%, in 17 of 24 patients (17). This treatment also relieved the motion sickness susceptibility of these subjects, predominantly women (18). In about 25 percent of the MdDS patients in the 2017 study, the initial improvement of 75 percent fell back to 50 percent after 1 month, but in that 50 percent the improvement was then maintained for a year or more (18).

It should be emphasized that this was the first and only successful treatment in patients who had been suffering for up to 15 to 20 years. The successful treatment of the MdDS has been carried on by Dai's colleague, Sergei Yakushin, Ph.D., Associate Professor of Neurology. Further experiments are being performed by Dr. Yakushin to determine if the improvement after the initial treatment can be stabilized by reducing the Velocity Storage time constant. This study is based on Dai's work that showed that motion sickness is reduced by reducing the vestibular time constant that underlies Velocity Storage (15). A comprehensive review of the MdDS can be found in a paper by Van Ombergen et al. (19). Additionally, a recent study indicated that the signs and symptoms of the MdDS could be produced by the movement of subjects on an oscillating platform that generated pitch, yaw and roll at low frequencies (20). This confirms that the MdDS is generated in the vestibular system.

## Conclusion

Dr. Dai's singular achievement in formulating the first successful treatment of the MdDS is remarkable, and I would like to have it noted by future physicians who use it to relieve its signs and symptoms. As such, I would like this treatment to be named the Dai treatment for the Mal de Debarquement Syndrome.

There is another reason to laud Dai's approach to treat MdDS. Namely, to my knowledge, this is the first medical treatment in the vestibular system that uses correction of a disordered rhythm and a return of a disordered orientation of the Eigenvector to gravity to cure a clinical condition. As such, it opens a new level of clinical treatment that could lead us to a deeper understanding of the interaction of the vestibular nuclei and the vestibulo-cerebellum, as well as to a better understanding of the neural basis of balance.

## Author Contributions

The author confirms being the sole contributor of this work and has approved it for publication.

### Conflict of Interest

The author declares that the research was conducted in the absence of any commercial or financial relationships that could be construed as a potential conflict of interest.
